# Transient Receptor Potential Melastatin-3 (TRPM3) Mediates Nociceptive-Like Responses in *Hydra vulgaris*

**DOI:** 10.1371/journal.pone.0151386

**Published:** 2016-03-14

**Authors:** Valentina Malafoglia, Lorenzo Traversetti, Floriano Del Grosso, Massimiliano Scalici, Filomena Lauro, Valeria Russo, Tiziana Persichini, Daniela Salvemini, Vincenzo Mollace, Massimo Fini, William Raffaeli, Carolina Muscoli, Marco Colasanti

**Affiliations:** 1 Department of Science, University of Roma Tre, Rome, Italy; 2 Institute for Research on Pain, ISAL-Foundation, Torre Pedrera (RN), Italy; 3 IRCCS San Raffaele Pisana, Rome, Italy; 4 Department of Pharmacological and Physiological Science, Saint Louis University School of Medicine, St Louis, United States of America; 5 IRC-FSH, Department of Health Science, University of ‘Magna Graecia’, Catanzaro, Italy; Universidade de São Paulo, BRAZIL

## Abstract

The ability of mammals to feel noxious stimuli lies in a heterogeneous group of primary somatosensory neurons termed nociceptors, which express specific membrane receptors, such as the Transient Receptor Potential (TRP) family. Here, we show that one of the most important nociceptive-like pathways is conserved in the freshwater coelenterate *Hydra vulgaris*, the most primitive organism possessing a nervous system. In particular, we found that *H*. *vulgaris* expresses TRPM3, a nociceptor calcium channel involved in the detection of noxious heat in mammals. Furthermore, we detected that both heat shock and TRPM3 specific agonist (*i*.*e*., pregnenolone sulfate) induce the modulation of the heat shock protein 70 (HSP70*)* and the nitric oxide synthase (NOS), two genes activated by TRP-mediated heat painful stimuli in mammals. As expected, these effects are inhibited by a TRPM3 antagonist (*i*.*e*., mefenamic acid). Interestingly, the TRPM3 agonist and heat shock also induce the expression of nuclear transcription erythroid 2-related factor (Nrf2) and superoxide dismutase (SOD), known markers of oxidative stress; noteworthy gene expression was also inhibited by the TRPM3 antagonist. As a whole, our results demonstrate the presence of conserved molecular oxidative/nociceptive-like pathways at the primordial level of the animal kingdom.

## Introduction

Pain study is becoming more and more preeminent because of the large epidemiology and the complexity of both the diagnosis and therapy [1]. Research in this field is still really difficult, because of the lack of economical and appropriate animal models in large scale [2, 3]. Exploiting non-standard animal models could have the potential to provide interesting insights into molecular mechanisms of nociception leading to pain-like behaviour.

In the last few years, we have been enormously interested in deepening down the evolutionary tree to find new simple animal models in order to highlight the basic nociceptive pathway [4, 5]. In particular, we had evidence of similar post-burn neuropathic pain in *Danio rerio*, commonly known as zebrafish [6].

This finding has encouraged us to examine the presence of conserved molecular pathways underlying nociception at the primordial level of the animal kingdom. The freshwater polyp *Hydra vulgaris* is an elementary organism belonging to the phylum of Cnidarian, class Hydrozoa, characterized by a saccular body structure composed by an apical (head) and a basal (foot) end [7]. The nervous system is extremely simple, consisting of few sensory neurons along a nerve net, through all the body [8]. Interestingly, a phylogenetical analysis of *Hydra* genome has demonstrated the existence of Transient Receptor Potential (TRP) channels, consisting on detectors and transducers ion channels [9]. In mammals, these Ca^2+^ permeable non-selective cation receptors mediate multiple noxious stimuli and physiologically elicit somatosensory responses to the environment [10]. In *Hydra*, at least 34 TRP channels have been identified, consisting in all the subfamily members, except for the vanilloid subfamily (TRPV), and five uncharacterized receptors [9].

Newly emerging evidence implicates the involvement of TRP channels, especially those of the melastatin subfamily (TRPM), in neuronal excitotoxicity processes via induction of nitro/oxidative stress [11]. In particular, nitric oxide (NO), superoxide anion (SO) and the potent nitro/oxidating agent peroxynitrite (PN) produced after N-methyl-D-aspartate receptor (NMDAR) activation, in turn activate TRPM channels resulting in Ca^2+^ influx and production of oxygen and nitrogen radicals [11]. This event leads to a vicious circle able to maintain high levels of PN that in turn maintains the nociceptive signalling [12, 13].

Earlier studies reported the critical role of oxidative stress during the development and maintenance of pain of several aetiologies, such as inflammatory central sensitization, hyperalgesia, chemotherapy-induced peripheral neuropathy (CIPN) and tolerance to morphine [12–14]. According to these data, we hypothesized that TRPs-related oxidative stress pathway could be a common defence *via* evolutionarily conserved from primitive organisms to human. Therefore, our aim was to evaluate the presence and the role of TRPM3-nociceptive/oxidative stress-like pathways in *H*. *vulgaris* following a thermal stimulus. Considering their over-expression after heat painful stimuli mediated by TRPs activation in mammals [15–19], we analyzed the expression of heat shock protein 70 (HSP70) and the nitric oxide synthase (NOS) genes. In addition, focusing on the emerging evidences showing the involvement of TRPs melastatin subfamily in oxidative stress pathway [11, 20], we chose the nuclear transcription erythroid 2-related factor (Nrf2), a known master regulator of the oxidative stress pathway [21, 22], and the superoxide dismutase (SOD), an Nrf2-depending enzyme [23].

## Materials and Methods

### Hydrae Husbandry

*Hydra vulgaris* Zurich strain was maintained in a 16h/8h light/dark cycle at 17°C in Hydra medium (1 mM calcium chloride, 0.1 mM sodium hydrogen carbonate, pH7) and fed once a week with Artemia nauplii, following standard husbandry protocols [24]. Budless polyps were selected for *in vivo* experiments and handled according to the guidelines of Roma3 University. Every effort was made to minimize the number of Hydrae used.

### Heat Shock (HS) Test

Animals were moved, with a specific net, from 17°C to 34°C Hydra medium beakers, for 1 minute, then placed again at 17°C and recovered in the incubator. Groups of 10 heated specimens were collected at specific time points after the heat shock (0, 0.5, 1.5, and 24 h). T = 0 was considered as a control. Animals were continuously monitored using an optical microscope, in order to reveal behavioural changes. For each time point, 10 specimens were processed for RNA extraction.

### Morphological and Behavioural Analysis

Polyp morphology and integrity was observed at optical microscopy, using a 32X magnification objective, before and after the HS test. Animals were collected in Petri dishes in Hydra medium and after a weak mechanical solicitation (needle) substrate adhesion, tentacles and body reactivity were analyzed as behavioural variables. All experiments were conducted with the experimenters blinded to treatment conditions.

### Treatment of *H*. *vulgaris* with Pregnenolone Sulfate and Mefenamic Acid

Currently, the most potent and selective available pharmacological tool to probe for biological roles of TRPM3 is the neuroactive steroid pregnenolone sulfate (PS), a selective agonist [25], and mefenamic acid (MFA), a selective and potent antagonist [26]. Animals were incubated with the PS (10 μM) and/or MFA (20 μM), up to 24h. PS and MFA right concentration for treatment was chosen after a dose-response test, based on scalar concentrations up to sublethal but efficient condition. According to our experimental observations, MFA needed 10 min of incubation before PS or HS treatment.

### RNA Extraction and cDNA Synthesis

Total RNA from *H*. *vulgaris* was extracted by using TRIzol^®^ Reagent (Life technologies Italia-Invitrogen, Monza, Italy). For each time point 10 hydrae were used. 1 μg of total RNA was reverse transcribed to cDNA by using GoTaq 2 Step RT qPCR System Protocol (Promega Italia Srl, Milan, Italy).

### Real Time PCRs (qPCRs)

PCR product quantification was calculated by applying SYBR-Green method. We used Master Mix from Promega. The primer pairs were chosen as described in S1 Table. Reactions were performed in Promega detection system, by using the following temperatures: pre-incubation 95°C, amplification at 95°C-60°C-72°C for 45 cycles, melting at 95°C-65°C-97°C, cooling at 37°C. Data are calculated relative to the internal housekeeping gene (β-actin).We applied the second derivative test, delta–delta Ct (2^-ΔΔCT^) method, choosing control samples to normalize our data.

### Membrane Receptor Extraction

*Hydra* cultures were homogenized in an ice-cold buffer (0.32 M sucrose, 100 μM sodium orthovanadate, 0.02 M glycerophosphate and 1% protease inhibitor cocktail (Sigma, Milan, Italy)) with a 1:3 w/v ratio. The homogenates were centrifuged at 800 g for 10min at 4°C. The resulting pellets were re-homogenized and centrifuged as before. The supernatants were combined and centrifuged at 12500 g at 4°C, for 30 minutes, to obtain a new pellet that was resuspended in homogenization buffer. Protein concentration was determined using the Pierce^™^ Bicinchoninic Acid (BCA) Protein Assay Kit (Thermo Scientific, Milan, Italy). Samples were stored at −80°C and were used to determine TRPM3 expression by western blot.

### Western Blot Analysis

Samples were loaded in 7.5% SDS-PAGE minigels (Bio-Rad Laboratories, Mi, Italy). After separating by SDS/PAGE proteins were transferred electrophoretically to nitrocellulose membranes (Bio-Rad).

Ponceau red (Sigma) staining was used to ensure successful proteins transfer. Membrane was blocked (2 h, room temperature) with 1% Bovine Serum Albumin (BSA)/1X TBS/0.1% Tween-20. Membrane was incubated with rabbit polyclonal anti-TRPM3 antibody (O/N, 4°C, 1:100 dilution; Alomone Labs, Jerusalem, Israel). After washing with TBS/T, the membrane was incubated with anti-rabbit horseradish peroxidase-conjugated secondary antibody (1 h, room temperature, 1:20000 dilution; Amersham, GE Healthcare Europe, Mi, Italy) and the specific complex was detected by an enhanced chemiluminescence detection system (ECL, Amersham). Quantitation of protein levels was then performed by densitometry using Image Quant 5.2 software by Molecular Dynamics.

### Immunofluorescence

Animals were relaxed and anaesthetized in 2% urethane for 2 minutes and then fixed in 4% paraformaldehyde for 24 hours at room temperature. After three washes with PBS, the animals were permeabilized with 0.1% Triton X-100 in PBS for 30 min, blocked with 1% BSA (w/v) in PBS for at least 20 min and incubated overnight at 4°C with TRPM3 (Alomone Labs) primary antibody diluted 1:200 in PBS/BSA0.1%. Several washes were followed by anti-rabbit antibody conjugated with FITC (Molecular Probes Alexa Fluor 488, Life Technologies Italia, Monza MB, Italy) incubation, diluted 1:200 for 1 hour. Excess antibody was removed by washing again. Sample were analyzed using confocal microscope (Leica TCS SP5) equipped with the software LAS AF version 1.6.3 (Leica Microsystems). Argon laser at 488 nm was activated for fluorochrome’s excitation and to acquire DIC (Differential Interference Contrast) images. Emission bandwidth: 500nm–535nm.

### Statistics

We statistically analyzed mRNA expression level for each gene through three technical repeats from three biological replicates, n = 3 (3 triplicates for each gene in a single experiment, for a total of 3 experiments) with one-way ANOVA (and nonparametric) assay, followed by Bonferroni's Multiple Comparison Test. Data are expressed as mean fold change compared with control (0 h) ± SEM.

## Results and Discussion

One of the biggest challenge is, doubtless, to find common features and predictable molecular pathways across different noxious stimuli with a consistent need of new innovative experimental models. To this end, in the last few years, we focused our attention on zebrafish, by proposing a similar post-burn neuropathic pain test in order to show the over-expression of pain markers genes after extreme thermal noxious stimuli, as happens in mammals [4–6]. Here, we consider the freshwater polyp *H*. *vulgaris* as an original animal model to search for conserved molecular nociceptive-like pathways after thermal stimuli.

**Fig 1:**
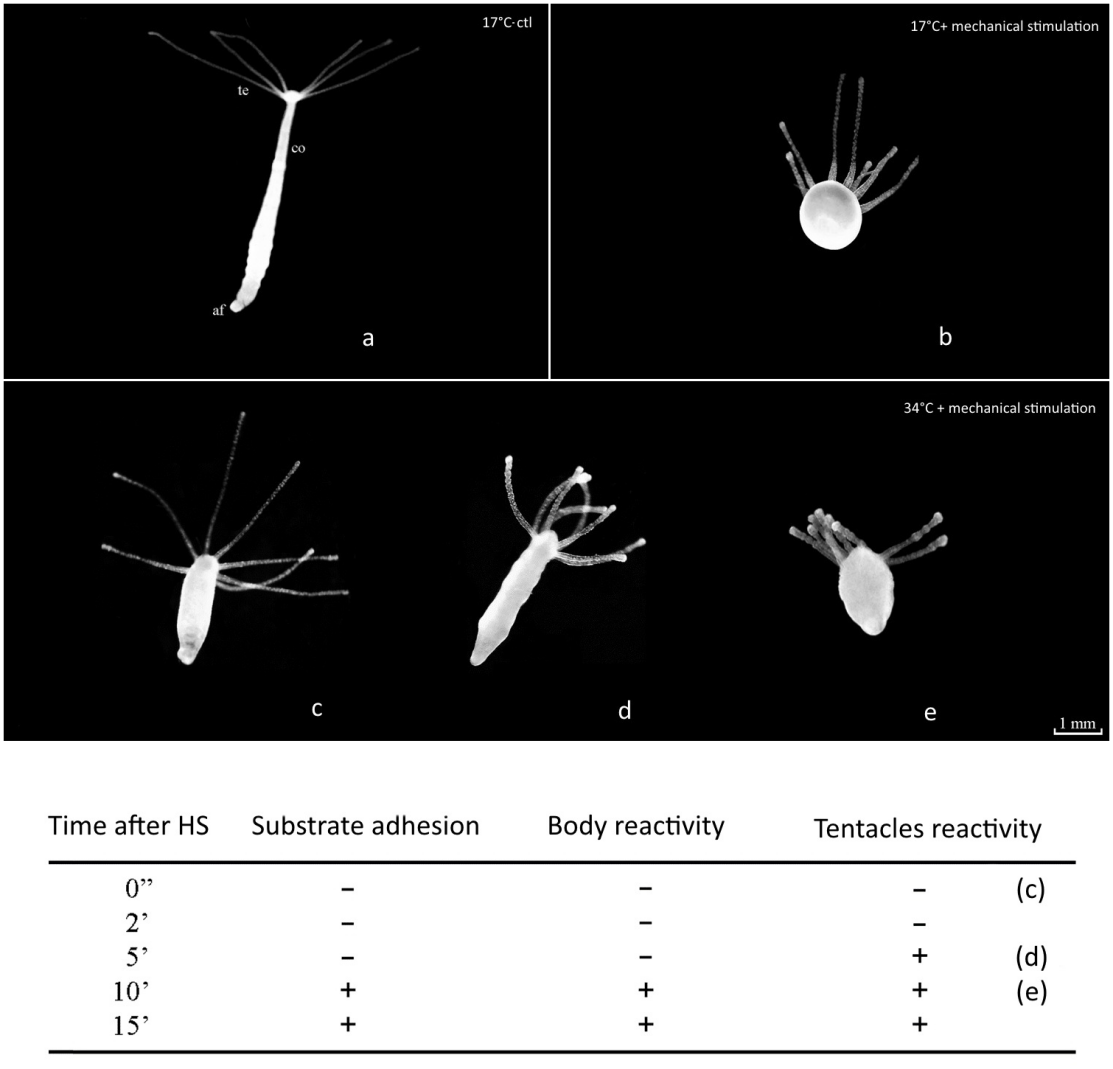
Common responses to mechanical solicitations in untreated (17°C) and HS-treated (34°C) animals. a) untreated polyp without mechanical solicitations: open tentacles, elongated body and foot adhered to the substrate; b) untreated polyp after mechanical solicitation: contraction of body and tentacles; c) HS-treated polyp followed by mechanical solicitation: open tentacles, elongated body and lack of adhesion to the substrate; d) HS-treated polyp was placed at 17°C for 5 min before mechanical solicitations: partial recovery of functions (e.g., tentacles reactivity); e) HS-treated polyp was placed at 17°C for 10 min before mechanical solicitations: total recovery of functional features. The table shows physiological variables analyzed during the HS time points; + means responsiveness,—means loss of responsiveness. Te: tentacles; af: adhesive foot; co: column.

Firstly, we processed animals through a heat shock (HS) test, starting from the physiological temperature (17°C) and going on, by increasing 4°C for each observation. We found 34°C as extreme sublethal efficient temperature to set up our analysis. Thus, we verified morphological and behavioural variables of specimens at 34°C, by monitoring common responses to weak mechanical solicitation, consisting in immediate contraction of both tentacles and body. As shown in Fig 1, heat shocked polyps (1 minute at 34°C) showed time-dependent modifications after mechanical stimulation, by resulting in instant inability to contract body and tentacles as well as loss of adhesion to the substrate, followed by recovery of physiological attitude.

In recent years, evidence has been accumulated regarding a relevant role of TRP channels in thermosensation. TRPs family is an evolutionarily conserved group of cation channels, of which a subset (*i*.*e*., the thermoTRPs) is known as thermoreceptors from flies to humans. ThermoTRPs fall into four basic classes: warm receptors which respond to innocuous (moderate) warming, cool receptors to innocuous cooling, high-temperature receptors to noxious (damaging) heat, and low-temperature receptors to noxious cold [27]. In *Hydra*, all the TRP members have been described, except for the TRPV subfamily. An excellent candidate as a mediator of thermosensation in *Hydra* is TRPM3, a nociceptor channel involved in the detection of noxious heat [28]. In fact, TRPM3 has a fairly moderate temperature coefficient, where temperature-evoked current increases gradually at values >15–17°C (*i*.*e*., the natural temperature in which *H*. *vulgaris* lives) [28].

**Fig 2:**
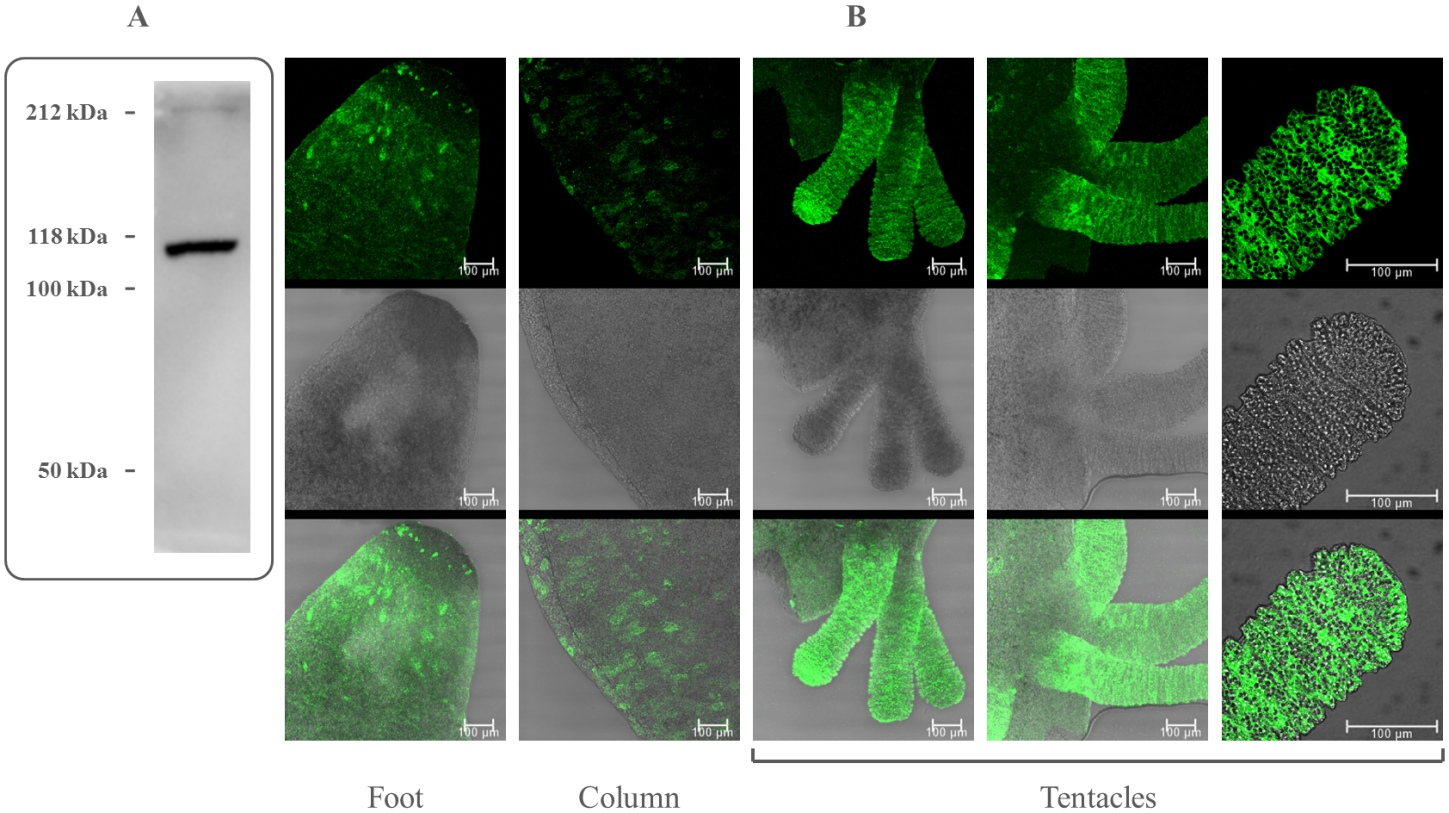
Identification, characterization and localization of *Hydra* TRPM3. (A) Expression of TRPM3 protein in whole body of *H*. *vulgaris*. Western blot is representative of six independent experiments. (B) Immunofluorescence of Hydra whole mounts with the anti-TRPM3 antibody. Signals for TRPM3 are clearly extracellular and appear mainly in tentacles and foot. Specimens were examined in the confocal microscope. Scale bars, 100 μm.

To reveal and localize the TRPM3 protein in *H*. *vulgaris*, we applied both Western blot analysis and immunofluorescence staining, respectively. The specific interaction between primary anti-TRPM3 antibody and *Hydra* lysates gave a single band, thus evidencing the presence of a TRPM3-like protein in the freshwater coelenterate (Fig 2A). To localize TRPM3 in tissue, *Hydra* polyps were first anesthetized and then fixed with PFA, stained with the anti-TRPM3 antibody and examined in the confocal microscope. Fig 2B shows that the TRPM3-positive material was clearly extracellular and appeared mainly in tentacles and foot while the column was weakly stained.

All of the ancient metazoan TRP channels may have important physiological roles in sensory perception, since they are known to respond to external environment. For example, animals use their thermosensory systems to achieve optimal temperatures for growth and reproduction and to avoid damaging thermal extremes. Recent papers shed light onto the diversity of thermosensory adaptations throughout evolution, including changes in properties of TRP family (for a recent review see [29]).

We focused our efforts on the studying TRPM3 but the possible involvement of other TRP members in thermosensation cannot be excluded at the moment. In mammals, for example, TRPM3 is strictly linked to TRPV1 that is activated at temperatures >42°C (a lethal temperature for *Hydra*). Interestingly, *Hydra* lacks TRPV channels although a novel TRP subfamily member (TRPVL) has been described. TRPVL shares common structural features with TRPV, suggesting that these two channels may have similar functions [9]. In mammals, TRPM3 is structurally and functionally linked also to TRPM1 [30]. In this respect, TRPM1 and TRPM3 proteins have been demonstrated to form functional heteromultimeric channels [30]. In particular, TRPM1 participates in the formation of the pore and thereby alters the properties of these channels [30] and the pregnenolone sulfate (PS), the most potent known activator of TRPM3, is also able to activate TRPM1 [31].

Understanding the TRP features in the context of the life histories and habitats of cnidarians would be of great interest to demonstrate the importance of a nociceptive-like system in low invertebrates.

**Fig 3:**
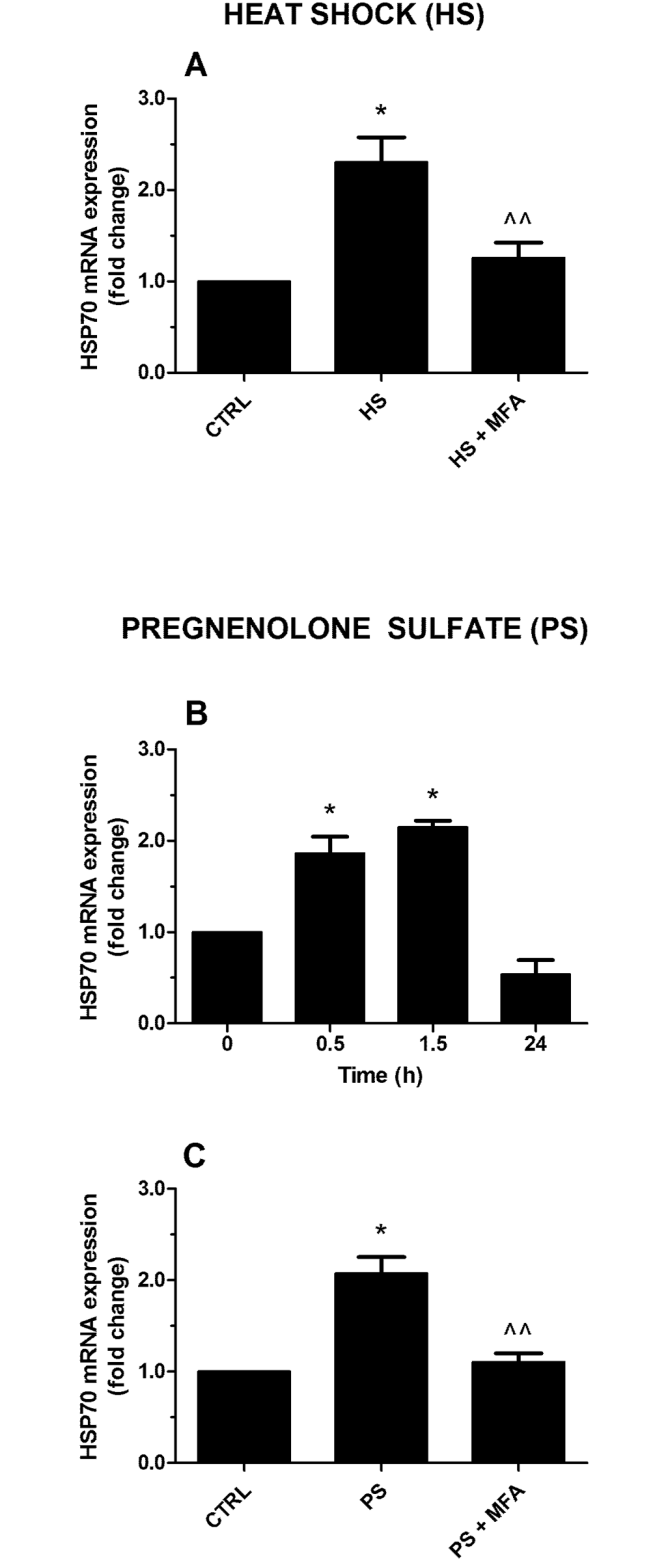
Role of TRPM3 on HSP70 mRNA expression in *H*. *vulgaris*. (A) A pretreatment for 10 min with the TRPM3 antagonist mefenamic acid (MFA; 20 μM) inhibits HSP70 gene expression induced by heat shock (HS; 34°C). (B) TRPM3 agonist pregnenolone sulfate (PS; 10 μM) increase HSP70 gene expression, the peak being observed after 1.5 h. (C) MFA (20 μM) inhibits HSP70 gene expression induced by PS (10 μM). Data are calculated relative to the internal housekeeping gene (β-actin) and are expressed as mean fold change compared with control (T = 0) ± SEM (n = 9). (A) * p≤0.01 vs CTRL and ^^ p≤0.05 vs HS; (B) * p≤0.01 vs 0; (C) * p≤0.01 vs CTRL and ^^ p≤0.05 vs PS.

In mammals, noxious thermal stimuli are followed by the activation and the over-expression of several marker genes, including HSP70 [15]. Moreover, the cellular heat shock response (*e*.*g*., HSP70 activation) induced by heat painful stimuli in mammalian epithelial cells has been associated with TRPs [15]. Note that HSP70 has been also cloned and functionally analyzed in different hydra species [32–35] and we confirmed that heat shock (HS) at 34°C for 1 minute increased HSP70 gene expression in *H*. *vulgaris*, the peak being observed after 1.5 h (see S1 Fig). To investigate the TRPM3 involvement in the HS-induced HSP70 expression in *H*. *vulgaris*, the TRPM3 selective antagonist mefenamic acid (MFA, 20 μM) has been used. In particular, we compared HSP70 mRNA modulation after HS (1.5 h) in two distinct groups of specimens: 1) HS and 2) MFA-preincubated HS polyps. We considered polyps at T = 0 as a control. Real time PCRs showed HSP70 ~2.5 folds of activation, as expected, in HS group (p≤0.01) and a reduction of expression, close to the control, in the second group treated with MFA (p≤0.05) (Fig 3A). To confirm TRPM3 involvement in the thermal noxious pathway, the specific agonist of the receptor, pregnenolone sulfate, has been employed [31]. In particular, it has been shown that PS-induced activation of TRPM3 evokes pain like behaviour in mice [28]. We incubated polyps with PS (10 μM) and collected animals at specific time points (0.5, 1.5, 24 h, and used T = 0 as a control) for molecular analysis. According to the HS results, HSP70 mRNA higher expression has been detected at 1.5 h in the PS treated polyps (~2 folds; p≤ 0.01) (Fig 3B). At the same time point, we repeated the test after PS and MFA pre-incubation, to validate TRPM3 implication in the process. Real time data showed loss of HSP70 mRNA modulation after PS-MFA co-treatment (p≤0.05) (Fig 3C).

In the last years, the activation of the TRP channels has been correlated with nitro-oxidative stress in mammalian nervous system. TRPA1, for example, is sensitive for large series of reactive products of oxidative and nitrative stress in models of different types of pain, including inflammatory, neuropathic pain and migraine [36]. In mammals, considerable evidence correlated oxidative stress to pathologic pain, determining nitro-oxidative species (e.g., NO, SO, PN) as a group of molecules capable of oxidative, nitrosative, and nitrative activities implicated in persistent pain states [11–14]. Constant nociceptive stimulation activates the SO-generating enzyme NADPH (nicotinamide adenine dinucleotide phosphate) oxidase and the NO-producing NOS. The interaction between SO and NO leads to the production of PN that acts as a potent pro-inflammatory nitro-oxidative species and a critical signaling molecule in the development of peripheral and central sensitization associated with pain of several etiologies [14].

The presence of NO pathway has been well demonstrated in Cnidaria, including *H*. *vulgaris* [37]. Our precedent studies have shown a NO involvement both in the feeding response [38] and in the regeneration processes of the head [39]. We have also demonstrated the presence of a calcium-dependent but calmodulin-independent NOS isoform [40]. Moreover, there is an existing body of literature characterizing the induction of NO pathway by heat stress in several cnidarian species. However, only host cells of symbiotic cnidarians (with the intracellular Dinoflagellate) produce *in hospite* NO during thermal stress [41]. NO produced during exposure to elevated temperature mediates a process known as cnidarian bleaching [42, 43], through a caspase-mediated pathway [44]. However, nothing is still known about NOS modulation neither by heat stress nor by TRP activation in an asymbiotic cnidarian (e.g. *Hydra*).

**Fig 4:**
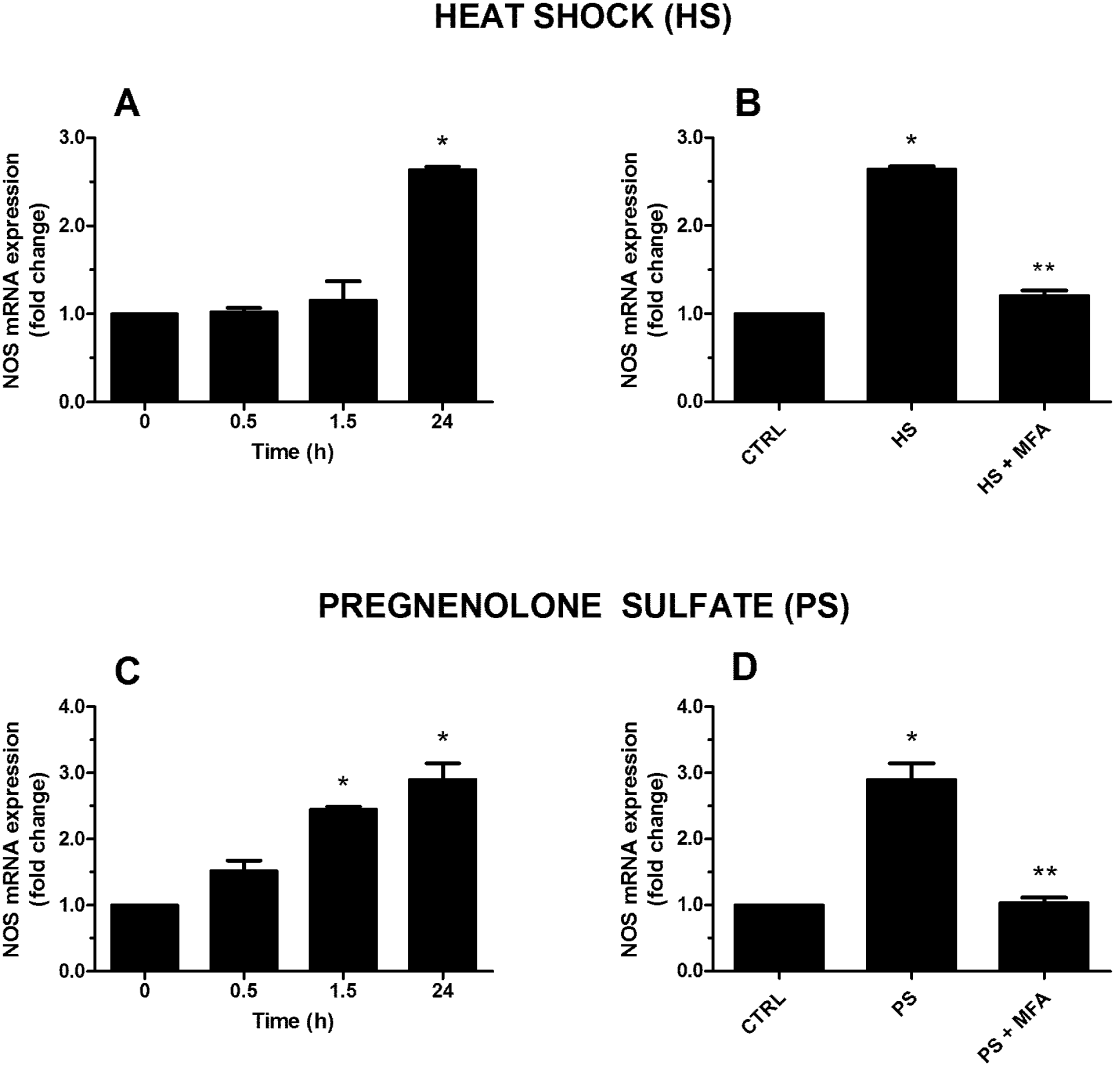
Effect of heat shock (HS; 34°C) and pregnenolone sulfate (PS; 10 μM) on NOS gene expression in *H*. *vulgaris*. HS (A) and PS (C) induce an increase of NOS mRNA expression with a maximal effect at 24 h. When animals are pretreated with mefenamic acid (MFA; 20 μM), NOS expression induced by HS (B) or PS (D) is abolished. Data are calculated relative to the internal housekeeping gene (β-actin) and are expressed as mean fold change compared with control (T = 0) ± SEM (n = 9). (A) * p≤0.01 vs 0; (B) * p≤0.01 vs CTRL and ** p≤0.01 vs HS; (C) * p≤0.01 vs 0; (D) * p≤0.01 vs CTRL and ** p≤0.01 vs PS.

Thus, we decided to test TRPM3 involvement in thermal noxious-like pathways in *H*. *vulgaris*, by analysing NOS mRNA over-expression after the HS (34°C, 1 min) at specific time points (0.5, 1.5, and 24 h, and used T = 0 as a control). We observed that the exposure of specimens to HS caused a marked increase of NOS gene expression at 24 h (~2.5 folds; p≤0.01) (Fig 4A). This effect was inhibited by MFA (20 μM) (p≤0.01) (Fig 4B), demonstrating a role of TRPM3 in this process. Again, to confirm TRPM3 recruitment, we used the specific receptor agonist PS (10 μM). When polyps were incubated with PS, an increase of NOS gene expression at 24 h was observed (p≤0.01) (Fig 4C), an effect being inhibited following MFA pre-incubation (p≤0.01) (Fig 4D) comparable to what observed with HS treatment. Through our experimental model, here we observed that thermal TRPM3 modulation contribute to NOS mRNA expression, thus resembling a nociceptive-like response. However, a direct effect of temperature on the catalytic activity of NOSs cannot be excluded [45].

Previously, SOD has been used as molecular biomarkers for assessing oxidative stress in *H*. *vulgaris*. Evidence exists that SOD is one of the major enzymes responsible for counteracting oxidative stress associated with toxaphene exposure in *H*. *magnipapillata* [46]. Furthermore, it has been reported that the expression patterns of the MnSOD and CuZnSOD mRNA were induced after stressor exposure, including heat treatment [23]. SOD activity and/or expression can be induced in corals by elevated temperatures and ultraviolet (UV) radiation, and these levels have been used as stress biomarkers in corals [47].

**Fig 5:**
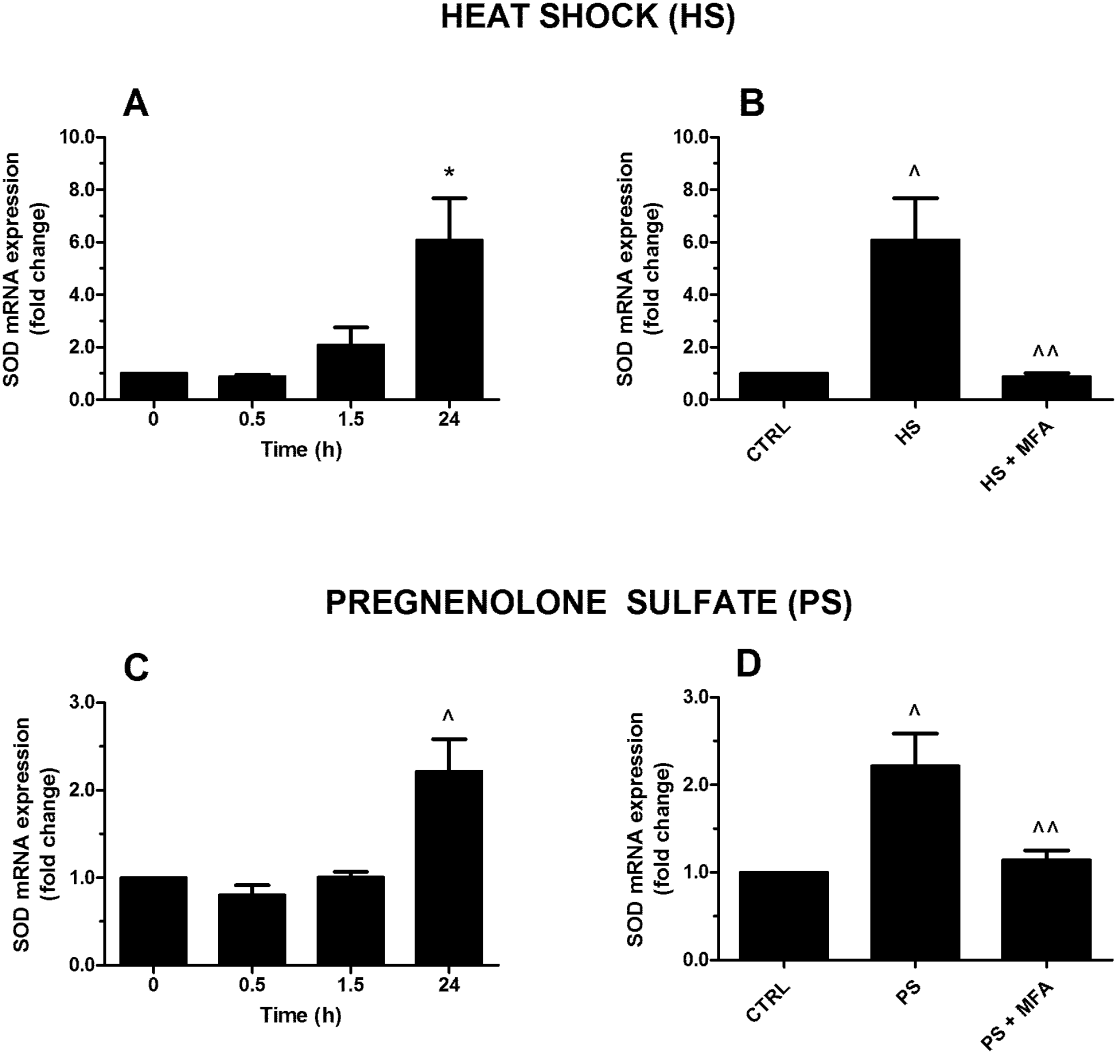
Effect of heat shock (HS; 34°C) and pregnenolone sulfate (PS; 10 μM) on HyCuZnSOD mRNA expression in *H*. *vulgaris*. Both HS (A) and TRPM3 agonist PS (C) induce an increase of SOD mRNA level, the maximal peak being observed after 24 h. A pretreatment with the TRPM3 antagonist mefenamic acid (MFA; 20 μM) inhibits the effect relative to HS (B) or PS (D). Data are calculated relative to the internal housekeeping gene (β-actin) and are expressed as mean fold change compared with control (0 h) ± SEM (n = 9). (A) * p≤0.01 vs 0; (B) ^ p≤0.05 vs CTRL and ^^ p≤0.05 vs HS; (C) ^ p≤0.05 vs 0; (D) ^ p≤0.05 vs CTRL and ^^ p≤0.05 vs PS.

Here, we analyzed the involvement of TRPM3 activation in CuZnSOD mRNA expression induced by HS and PS treatment in *H*. *vulgaris*. In particular, the exposure of polyps to HS increased SOD mRNA expression at 24 h with ~8 folds of change (p≤0.01) (Fig 5A) and this effect was abolished by 20 μM MFA (p≤0.05) (Fig 5B), thereby suggesting that TRPM3 mediated HS-induced SOD expression. When polyps were incubated with 10 μM PS, a time-dependent increase of SOD gene expression at 24 h was observed with ~2.5 folds of change (p≤0.05) (Fig 5C) and this effect was inhibited by 20 μM MFA (p≤0.05), thus confirming an involvement of TRPM3 in these mechanisms (Fig 5D).

During the oxidative stress response in vertebrates, a major regulator of detoxifying/antioxidant enzymes (e.g. SOD) is the nuclear factor erythroid 2 related factor 2 (Nrf2), which migrates to the nucleus where it acts as a transcription factor. New data reported Nrf2 activation due to a heat treatment in mice [48]. A noxious injury-induced transcriptomic analysis showed a transient up-regulation of Nrf2 gene in *Hydra* [49].

**Fig 6:**
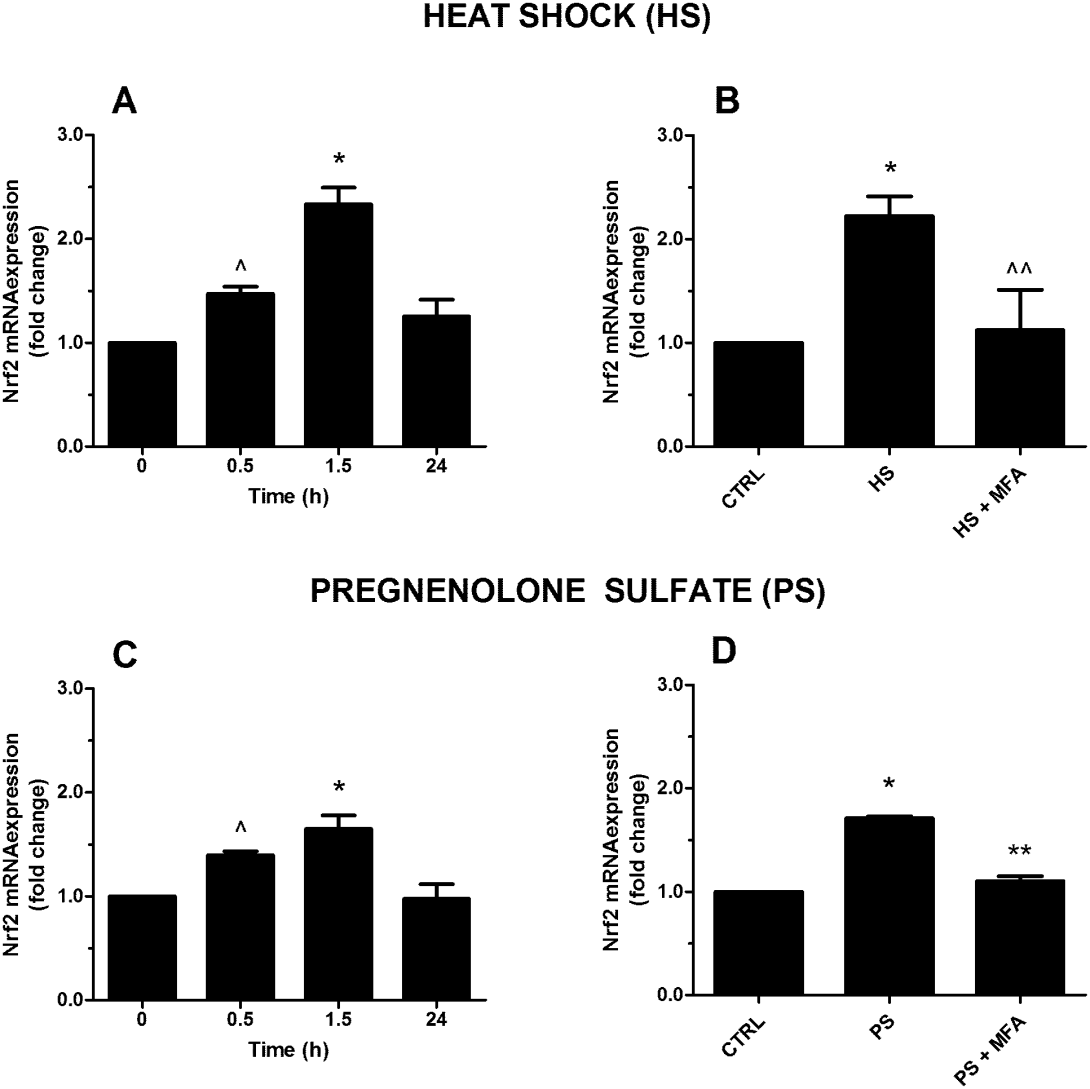
Action of heat shock (HS; 34°C) and pregnenolone sulfate (PS; 10 μM) on Nrf2 mRNA expression in *H*. *vulgaris*. HS (A) and the TRPM3 agonist PS (C) time-dependently induce Nrf2 with a peak observed after 1.5 h. A pretreatment for 10 min with the TRPM3 antagonist mefenamic acid (MFA; 20 μM) inhibits the effect relative to both HS (B) and PS (D). Data are calculated relative to the internal housekeeping gene (β-actin) and are expressed as mean fold change compared with control (0 h) ± SEM (n = 9). (A) * p≤0.01 vs 0 and ^ p≤0.05 vs 0; (B) * p≤0.01 vs CTRL and ^^ p≤0.05 vs HS; (C) * p≤0.01 vs 0 and ^ p≤0.05 vs 0; (D) * p≤0.01 vs CTRL and ** p≤0.01 vs PS.

In this respect, we detected Nrf2 modulation at specific time points (0.5, 1.5, and 24 h, and used T = 0 as a control) through HS test. During HS test, Nrf2 mRNA expression increased, reaching the higher point of expression at 1.5 h, with ~2.5 folds of change (p≤0.01) (Fig 6A). We demonstrated TRPM3 involvement by monitoring the inhibition of Nrf2 transcription by using 20 μM MFA (p≤0.05) (Fig 6B). We went on in confirming TRPM3 role in the process, by treating polyps with PS (10 μM) and by finding increased Nrf2 mRNA expression ~1.7 folds of change at 1.5 h (p≤0.01) (Fig 6C) and decreased Nrf2 transcription after PS/MFA treatment (p≤0.01) (Fig 6D). Taken together, our findings are consistent with the emerging evidences showing the involvement of TRPs, especially those of the melastatin subfamily, in the oxidative stress pathway during noxious stimuli. During the last decade, specific attention has been given to the TRPs family as a crucial mechanism for the transition from acute to chronic pain condition and as the primary transducing pathway by which nitro-oxidative stress could contribute to acute nociception, allodynia, and hyperalgesia. In particular, there is evidence that reactive oxygen and nitrogen species are responsible for the activation of nociceptors TRP channels resulting in Ca^2+^ influx. These events cause e positive feedback, in fact, NO can regulate TRPs directly via Cys *S*-nitrosylation or indirectly via cyclic GMP/protein kinase G-dependent phosphorylation [18].

## Conclusions

As a whole, we found that the exposition of *H*. *vulgaris* to heat induces the expression of HSP70 and NOS, two genes induced by TRP-mediated heat noxious stimuli in mammals [15, 18] as well as Nrf2 and SOD, known as markers of the oxidative stress during pain conditions [21, 50]. It appears that both of the effects are mediated by the modulation of TRPM3, a receptor involved in the detection of noxious heat also in mammals, thus suggesting a nociceptive-like response at the primordial level of the animal kingdom that involves the nitro-oxidative pathway. However, whether and how the proposed mechanisms can cause nociception in *H*. *vulgaris* remains to be further investigated.

The presence of TRPs and its modulation, showed here, are intriguing and revolutionary data demonstrating the importance of a nociceptive-like system in low invertebrates.

## Supporting Information

S1 Fig Effect of heat shock (HS; 34°C) on HSP70 mRNA expression in *H*. *vulgaris*. HSP70 mRNA transcription after thermal stimulation was quantitatively analysed by comparing it to actin expression, as housekeeping gene. We tested up to 3 time points (expressed hours after test): 0.5, 1.5, and 24 h, and used T = 0 as a control. After 0.5 h post-treatment, HSP70 mRNA quantity increased ~2 folds, compared to the control (p≤0.05) and reached its maximum values of ~2.5 folds after 1.5 h (p≤0.01), and it comes back to a physiological level at 24 h. * p≤0.01 vs 0 and ^ p≤0.05 vs 0.
(PDF)

S1 Table Sequences of primers used for RT-PCR and real-time quantitative PCR to quantify levels of H. vulgaris transcripts that encode proteins involved in the nociceptive-like response. (PDF)

## References

[1] Raffaeli W, Andruccioli J, Florindi S, Ferioli I, Monterubbianesi MC, Sarti D, et al Qualitative pain classification in hospice and pain therapy unit. Am J Hosp Palliat Care. 2012;29(8):604–9. 10.1177/1049909111435810 22310024

[2] Campana G, Sarti D, Spampinato S, Raffaeli W. Long-term intrathecal morphine and bupivacaine upregulate MOR gene expression in lymphocytes. Int Immunopharmacol. 2010;10(9):1149–52. 10.1016/j.intimp.2010.06.016 20609402

[3] Cargnin S, Magnani F, Viana M, Tassorelli C, Mittino D, Cantello R, et al An opposite-direction modulation of the COMT Val158Met polymorphism on the clinical response to intrathecal morphine and triptans. J Pain. 2013;14(10):1097–106. 10.1016/j.jpain.2013.04.006 23773341

[4] Gigliuto C, De GM, Malafoglia V, Raffaeli W, Compagnone C, Visai L, et al Pain assessment in animal models: do we need further studies? JPain Res. 2014;7:227–36. 24855386 10.2147/JPR.S59161 PMC4020878

[5] Malafoglia V, Bryant B, Raffaeli W, Giordano A, Bellipanni G. The zebrafish as a model for nociception studies. JCell Physiol. 2013;228(10):1956–66. 23559073 10.1002/jcp.24379

[6] Malafoglia V, Colasanti M, Raffaeli W, Balciunas D, Giordano A, Bellipanni G. Extreme thermal noxious stimuli induce pain responses in zebrafish larvae. JCell Physiol. 2014;229(3):300–8. 23929528 10.1002/jcp.24447 PMC4106021

[7] Chapman JA, Kirkness EF, Simakov O, Hampson SE, Mitros T, Weinmaier T, et al The dynamic genome of Hydra. Nature. 2010;464(7288):592–6. 10.1038/nature08830 20228792 PMC4479502

[8] Technau U, Steele RE. Evolutionary crossroads in developmental biology: Cnidaria. Development. 2011;138(8):1447–58. 10.1242/dev.048959 21389047 PMC3062418

[9] Peng G, Shi X, Kadowaki T. Evolution of TRP channels inferred by their classification in diverse animal species. MolPhylogenetEvol. 2015;84:145–57. 10.1016/j.ympev.2014.06.016 24981559

[10] Mickle AD, Shepherd AJ, Mohapatra DP. Sensory TRP Channels: The Key Transducers of Nociception and Pain. ProgMolBiolTranslSci. 2015;131:73–118. 10.1016/bs.pmbts.2015.01.002 PMC5903472 25744671

[11] Forder JP, Tymianski M. Postsynaptic mechanisms of excitotoxicity: Involvement of postsynaptic density proteins, radicals, and oxidant molecules. Neuroscience. 2009;158(1):293–300. 10.1016/j.neuroscience.2008.10.021 19041375

[12] Muscoli C, Mollace V, Wheatley J, Masini E, Ndengele M, Wang ZQ, et al Superoxide-mediated nitration of spinal manganese superoxide dismutase: a novel pathway in N-methyl-D-aspartate-mediated hyperalgesia. Pain. 2004;111(1–2):96–103. 15327813 10.1016/j.pain.2004.06.004

[13] Muscoli C, Dagostino C, Ilari S, Lauro F, Gliozzi M, Bardhi E, et al Posttranslational nitration of tyrosine residues modulates glutamate transmission and contributes to N-methyl-D-aspartate-mediated thermal hyperalgesia. MediatorsInflamm. 2013;2013:950947. 10.1155/2013/950947 PMC3705874 23864769

[14] Doyle T, Chen Z, Muscoli C, Bryant L, Esposito E, Cuzzocrea S, et al Targeting the overproduction of peroxynitrite for the prevention and reversal of paclitaxel-induced neuropathic pain. JNeurosci. 2012;32(18):6149–60. 22553021 10.1523/JNEUROSCI.6343-11.2012 PMC3752044

[15] Bromberg Z, Goloubinoff P, Saidi Y, Weiss YG. The membrane-associated transient receptor potential vanilloid channel is the central heat shock receptor controlling the cellular heat shock response in epithelial cells. PLoSOne. 2013;8(2):e57149. 10.1371/journal.pone.0057149 PMC3584136 23468922

[16] Hervera A, Negrete R, Leanez S, Martin-Campos JM, Pol O. The spinal cord expression of neuronal and inducible nitric oxide synthases and their contribution in the maintenance of neuropathic pain in mice. PLoSOne. 2010;5(12):e14321. 10.1371/journal.pone.0014321 PMC3001461 21179208

[17] Keilhoff G, Schroder H, Peters B, Becker A. Time-course of neuropathic pain in mice deficient in neuronal or inducible nitric oxide synthase. NeurosciRes. 2013;77(4):215–21. 10.1016/j.neures.2013.08.008 24008126

[18] Leonelli M, Martins DO, Britto LR. Retinal cell death induced by TRPV1 activation involves NMDA signaling and upregulation of nitric oxide synthases. Cell MolNeurobiol. 2013;33(3):379–92. 10.1007/s10571-012-9904-5 PMC11497882 23324998

[19] Sung CS, Wen ZH, Chang WK, Ho ST, Tsai SK, Chang YC, et al Intrathecal interleukin-1beta administration induces thermal hyperalgesia by activating inducible nitric oxide synthase expression in the rat spinal cord. Brain Res. 2004;1015(1–2):145–53. 15223378 10.1016/j.brainres.2004.04.068

[20] Miyake T, Shirakawa H, Kusano A, Sakimoto S, Konno M, Nakagawa T, et al TRPM2 contributes to LPS/IFNgamma-induced production of nitric oxide via the p38/JNK pathway in microglia. BiochemBiophysResCommun. 2014;444(2):212–7. 10.1016/j.bbrc.2014.01.022 24462864

[21] Nguyen T, Nioi P, Pickett CB. The Nrf2-antioxidant response element signaling pathway and its activation by oxidative stress. JBiolChem. 2009;284(20):13291–5. 10.1074/jbc.R900010200 PMC2679427 19182219

[22] Vriend J, Reiter RJ. The Keap1-Nrf2-antioxidant response element pathway: a review of its regulation by melatonin and the proteasome. MolCell Endocrinol. 2015;401:213–20. 10.1016/j.mce.2014.12.013 25528518

[23] Dash B, Metz R, Huebner HJ, Porter W, Phillips TD. Molecular characterization of two superoxide dismutases from Hydra vulgaris. Gene. 2007;387(1–2):93–108. 17150313 10.1016/j.gene.2006.08.020 PMC1855153

[24] Loomis WF. The cultivation of hydra under controlled conditions. Science. 1953;117(3047):565–6. 13056619 10.1126/science.117.3047.565

[25] Lesch A, Rubil S, Thiel G. Activation and inhibition of transient receptor potential TRPM3-induced gene transcription. BrJPharmacol. 2014;171(10):2645–58. 10.1111/bph.12524 PMC4009006 24895737

[26] Klose C, Straub I, Riehle M, Ranta F, Krautwurst D, Ullrich S, et al Fenamates as TRP channel blockers: mefenamic acid selectively blocks TRPM3. BrJPharmacol. 2011;162(8):1757–69. 10.1111/j.1476-5381.2010.01186.x PMC3081119 21198543

[27] Laing RJ, Dhaka A. ThermoTRPs and Pain. Neuroscientist. 2015. 10.1177/1073858414567884 PMC4510032 25608689

[28] Vriens J, Owsianik G, Hofmann T, Philipp SE, Stab J, Chen X, et al TRPM3 is a nociceptor channel involved in the detection of noxious heat. Neuron. 2011;70(3):482–94. 10.1016/j.neuron.2011.02.051 21555074

[29] Gracheva E and Bagriantsev SN. Evolutionary adaptation to thermosensation. Curr. Opinion Neurobiol. 2015;34:67–73. 10.1016/j.conb.2015.01.021 25698346

[30] Lambert S, Drews A, Rizun O, Wagner TF, Lis A, Mannebach S, et al Transient receptor potential melastatin 1 (TRPM1) is an ion-conducting plasma membrane channel inhibited by zinc ions. J Biol Chem. 2011;286(14):12221–33. 10.1074/jbc.M110.202945 21278253 PMC3069426

[31] Harteneck C. Pregnenolone sulfate: from steroid metabolite to TRP channel ligand. Molecules. 2013;18(10):12012–28. 10.3390/molecules181012012 24084011 PMC6270300

[32] Ambrosone A, del PP, Marchesano V, Parak WJ, de la Fuente JM, Tortiglione C. Gold nanoprisms for photothermal cell ablation in vivo. Nanomedicine(Lond). 2014;9(13):1913–22. 24877877 10.2217/nnm.14.100

[33] Bosch TC, Krylow SM, Bode HR, Steele RE. Thermotolerance and synthesis of heat shock proteins: these responses are present in Hydra attenuata but absent in Hydra oligactis. ProcNatlAcadSciUSA. 1988;85(21):7927–31. 10.1073/pnas.85.21.7927 PMC282326 3186697

[34] Brennecke T, Gellner K, Bosch TC. The lack of a stress response in Hydra oligactis is due to reduced hsp70 mRNA stability. EurJBiochem. 1998;255(3):703–9. 10.1046/j.1432-1327.1998.2550703.x 9738911

[35] Gellner K, Praetzel G, Bosch TC. Cloning and expression of a heat-inducible hsp70 gene in two species of Hydra which differ in their stress response. EurJBiochem. 1992;210(3):683–91. 10.1111/j.1432-1033.1992.tb17469.x 1483453

[36] Nassini R, Materazzi S, Benemei S, Geppetti P. The TRPA1 channel in inflammatory and neuropathic pain and migraine. RevPhysiol BiochemPharmacol. 2014;167:1–43. 10.1007/112_2014_18 24668446

[37] Colasanti M, Persichini T, Venturini G. Nitric oxide pathway in lower metazoans. NitricOxide. 2010;23(2):94–100. 10.1016/j.niox.2010.05.286 20638951

[38] Colasanti M, Lauro GM, Venturini G. NO in hydra feeding response. Nature. 1995;374(6522):505. 10.1038/374505a0 7535383

[39] Colasanti M, Mazzone V, Mancinelli L, Leone S, Venturini G. Involvement of nitric oxide in the head regeneration of Hydra vulgaris. NitricOxide. 2009;21(3–4):164–70. 10.1016/j.niox.2009.07.003 19635580

[40] Colasanti M, Venturini G, Merante A, Musci G, Lauro GM. Nitric oxide involvement in Hydra vulgaris very primitive olfactory-like system. JNeurosci. 1997;17(1):493–9. 8987773 10.1523/JNEUROSCI.17-01-00493.1997 PMC6793700

[41] Safavi-Hemami H, Young ND, Doyle J, Llewellyn L, Klueter A. Characterisation of nitric oxide synthase in three cnidarian-dinoflagellate symbioses. PLoSOne. 2010;5(4):e10379. 10.1371/journal.pone.0010379 PMC2861001 20442851

[42] Bouchard JN, Yamasaki H. Heat stress stimulates nitric oxide production in Symbiodinium microadriaticum: a possible linkage between nitric oxide and the coral bleaching phenomenon. Plant Cell Physiol. 2008;49(4):641–52. 10.1093/pcp/pcn037 18308760

[43] Perez S, Weis V. Nitric oxide and cnidarian bleaching: an eviction notice mediates breakdown of a symbiosis. JExpBiol. 2006;209(Pt 14):2804–10. 10.1242/jeb.02309 16809471

[44] Hawkins TD, Bradley BJ, Davy SK. Nitric oxide mediates coral bleaching through an apoptotic-like cell death pathway: evidence from a model sea anemone-dinoflagellate symbiosis. FASEB J. 2013;27(12):4790–8. 10.1096/fj.13-235051 23934282

[45] Venturini G, Colasanti M, Fioravanti E, Bianchini A, Ascenzi P. Direct effect of temperature on the catalytic activity of nitric oxide synthases types I, II, and III. Nitric Oxide. 1999;3(5):375–82. 10534441 10.1006/niox.1999.0250

[46] Woo S, Lee A, Won H, Ryu JC, Yum S. Toxaphene affects the levels of mRNA transcripts that encode antioxidant enzymes in Hydra. Comp BiochemPhysiol CToxicolPharmacol. 2012;156(1):37–41. 10.1016/j.cbpc.2012.03.005 22498080

[47] Downs CA, Mueller E, Phillips S, Fauth JE, Woodley CM. A molecular biomarker system for assessing the health of coral (Montastraea faveolata) during heat stress. MarBiotechnol(NY). 2000;2(6):533–44. 10.1007/s101260000038 14961177

[48] Li Y, Cao Y, Wang F, Li C. Scrotal heat induced the Nrf2-driven antioxidant response during oxidative stress and apoptosis in the mouse testis. Acta Histochem. 2014;116(5):883–90. 10.1016/j.acthis.2014.02.008 24698288

[49] Wenger Y, Buzgariu W, Reiter S, Galliot B. Injury-induced immune responses in Hydra. SeminImmunol. 2014;26(4):277–94. 10.1016/j.smim.2014.06.004 25086685

[50] Rosa AO, Egea J, Lorrio S, Rojo AI, Cuadrado A, Lopez MG. Nrf2-mediated haeme oxygenase-1 up-regulation induced by cobalt protoporphyrin has antinociceptive effects against inflammatory pain in the formalin test in mice. Pain. 2008;137(2):332–9. 17964723 10.1016/j.pain.2007.09.015

