# Supplementary material for: Transient Receptor Potential Melastatin-3 (TRPM3) Mediates Nociceptive-Like Responses in Hydra vulgaris
Source: PLoS One. 2016 Mar 14;11(3):e0151386. doi: 10.1371/journal.pone.0151386 (PMC4790967; doi:10.1371/journal.pone.0151386)
Supplement: S1 Fig — HSP70 mRNA transcription after thermal stimulation was quantitatively analysed by comparing it to actin expression, as housekeeping gene. We tested up to 3 time points (expressed hours after test): 0.5, 1.5, and 24 h, and used T = 0 as a control. After 0.5 h post-treatment, HSP70 mRNA quantity increased ~2 folds, compared to the control (p≤0.05) and reached its maximum values of ~2.5 folds after 1.5 h (p≤0.01), and it comes back to a physiological level at 24 h. * p≤0.01 vs 0 and ^ p≤0.05 vs 0. (PDF) [file pone.0151386.s001.pdf]

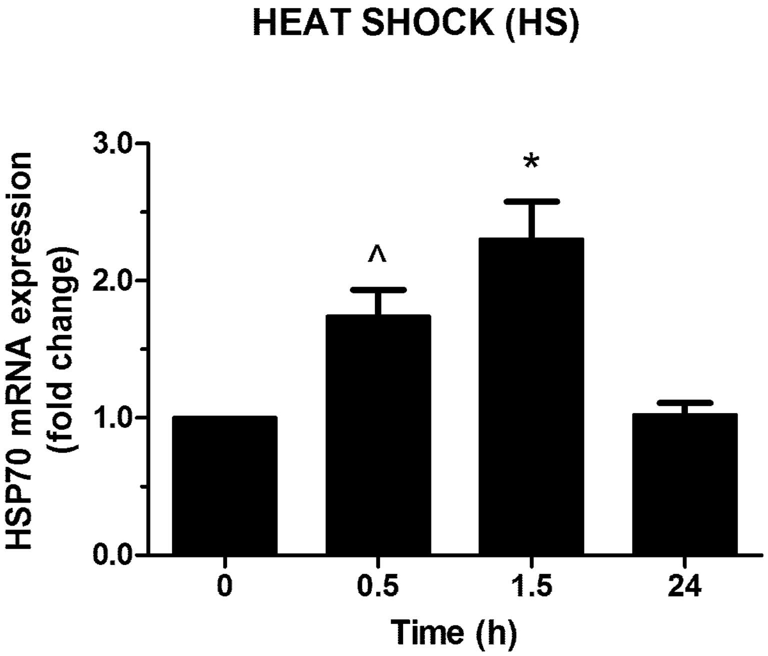

S1 Fig. Effect of heat shock (HS; 34°C) on HSP70 mRNA expression in *H. vulgaris*. HSP70 mRNA transcription after thermal stimulation was quantitatively analysed by comparing it to actin expression, as housekeeping gene. We tested up to 3 time points (expressed hours after test): 0.5, 1.5, and 24 h, and used T=0 as a control. After 0.5 h post-treatment, HSP70 mRNA quantity increased ~2 folds, compared to the control ($p \leq 0.05$) and reached its maximum values of ~2.5 folds after 1.5 h ($p \leq 0.01$), and it comes back to a physiological level at 24 h. * $p \leq 0.01$ vs 0 and ^ $p \leq 0.05$ vs 0.
