# Supplementary material for: Transient Receptor Potential Melastatin-3 (TRPM3) Mediates Nociceptive-Like Responses in Hydra vulgaris
Source: PLoS One. 2016 Mar 14;11(3):e0151386. doi: 10.1371/journal.pone.0151386 (PMC4790967; doi:10.1371/journal.pone.0151386)
Supplement: S1 Table — (PDF) [file pone.0151386.s002.pdf]

S1 Table. Sequences of primers used for RT-PCR and real-time quantitative PCR to quantify levels of *H. vulgaris* transcripts that encode proteins involved in the nociceptive-like response.

Gene	nucleotide sequence	Expected size (bp)
HSP70	forward 5’-ATG CGA AAC GAT TGA TTG GT-3’	114
	reverse 5’ACT CAA CTT GAA TCT TTG GC-3’	
NOS	forward 5’-TAT CAA GCA GCA GGT GTG AC- 3’	141
	reverse 5’-TAC AGA TCC AGA AAG CGG AG-3’	
CuZnSOD	forward 5'-TCAGTTTTGGGGATTATTCAGGTG-3'	280
	reverse 5’-CAAAACCACCGGAAATGCTGGA-3’	
Nrf2	forward 5-CTA GTA GAG TCA TTA TCT CC-3’	137
	reverse 5-AAA CTT GAA TCT GAC CTC TG-3’	
Actin	forward 5’-TCC TTG TAT GCT TCT GGT CG-3’	107
	reverse 5’-ATA ATG GCA TGG GGA AGA GC-3’	
